# Pharmacological Profiling of a Brugia malayi Muscarinic Acetylcholine Receptor as a Putative Antiparasitic Target

**DOI:** 10.1128/aac.01188-22

**Published:** 2023-01-05

**Authors:** Kendra J. Gallo, Nicolas J. Wheeler, Abdifatah M. Elmi, Paul M. Airs, Mostafa Zamanian

**Affiliations:** a Department of Pathobiological Sciences, University of Wisconsin—Madison, Madison, Wisconsin, USA

**Keywords:** *Brugia malayi*, *Caenorhabditis elegans*, GPCR, anthelmintics, antiparasitics, filariasis, helminths, muscarinic, nematode, parasitology

## Abstract

The diversification of anthelmintic targets and mechanisms of action will help ensure the sustainable control of nematode infections in response to the growing threat of drug resistance. G protein-coupled receptors (GPCRs) are established drug targets in human medicine but remain unexploited as anthelmintic substrates despite their important roles in nematode neuromuscular and physiological processes. Bottlenecks in exploring the druggability of parasitic nematode GPCRs include a limited helminth genetic toolkit and difficulties establishing functional heterologous expression. In an effort to address some of these challenges, we profile the function and pharmacology of muscarinic acetylcholine receptors in the human parasite Brugia malayi, an etiological agent of human lymphatic filariasis. While acetylcholine-gated ion channels are intensely studied as targets of existing anthelmintics, comparatively little is known about metabotropic receptor contributions to parasite cholinergic signaling. Using multivariate phenotypic assays in microfilariae and adults, we show that nicotinic and muscarinic compounds disparately affect parasite fitness traits. We identify a putative G protein-linked acetylcholine receptor of B. malayi (*Bma*-GAR-3) that is highly expressed across intramammalian life stages and adapt spatial RNA *in situ* hybridization to map receptor transcripts to critical parasite tissues. Tissue-specific expression of *Bma-gar-3* in Caenorhabditis elegans (body wall muscle, sensory neurons, and pharynx) enabled receptor deorphanization and pharmacological profiling in a nematode physiological context. Finally, we developed an image-based feeding assay as a reporter of pharyngeal activity to facilitate GPCR screening in parasitized strains. We expect that these receptor characterization approaches and improved knowledge of GARs as putative drug targets will further advance the study of GPCR biology across medically important nematodes.

## INTRODUCTION

Parasitic nematodes cause infectious diseases of poverty endemic to underdeveloped and exploited countries, accounting for the loss of over 8 million disability-adjusted life years (1). Current control mechanisms for helminth infections rely on mass drug administration (MDA) with a limited arsenal of drugs. Lymphatic filariasis (LF) is a neglected tropical disease caused by mosquito-transmitted nematodes (Wuchereria bancrofti, Brugia malayi, and Brugia timori) that migrate to and develop in human lymphatic systems (2). An estimated 50 million people currently have LF, with at least 36 million people suffering from chronic debilitating and highly stigmatizing conditions such as elephantiasis and hydrocele (3–6). The anthelmintics used for LF treatment are suboptimal; they do not kill adult-stage parasites and are contraindicated in regions coendemic for closely related parasites. Furthermore, the threat of anthelmintic resistance (7–13) underscores a recognized need for new drugs to treat vector- and soil-transmitted nematode infections in human and animal populations.

The current anthelmintics were primarily discovered using animal or whole-organism screening approaches (14, 15), and no new anthelmintics have been approved for human use in decades. Target-based approaches may provide an alternative route to screening validated molecular targets at much higher throughput (15–17), but bottlenecks derive from limited knowledge of basic parasite biology, a dearth of actionable targets, and difficulties in establishing reliable heterologous platforms for target expression and screening (16, 18). While ligand-gated ion channels (LGICs) receive warranted attention as the primary targets of existing anthelmintics, there is a need to diversify and pursue other druggable proteins critical to the physiology and survival of parasitic nematodes (9, 19–22).

G protein-coupled receptors (GPCRs) are highly druggable and are the targets of over one-third of all FDA-approved drugs in human medicine (23). Despite their recognition as lucrative targets (24–29), helminth GPCRs have yet to be effectively exploited as anthelmintic substrates. Studies of GPCRs and their ligands in free-living nematodes show that this receptor family is involved in a range of important physiological processes (30–34). Biogenic amines and neuropeptides elicit phenotypes of interest in free-living (30, 31, 35, 36) and parasitic nematodes (26, 37–39), many of which are likely mediated by metabotropic receptors. However, there is little data on the localization and function of parasitic nematode GPCRs, and pharmacological data are scant, partly due to difficulties in establishing reliable heterologous expression in single-cell systems (18, 40, 41). Methods to characterize GPCRs in less tractable parasite species will better enable the prioritization of new receptor leads with host-divergent pharmacological profiles that can be selectively targeted.

Acetylcholine (ACh) and its receptor targets are essential for growth, development, and neuromuscular function in the clade V model nematode Caenorhabditis elegans (42, 43). The contribution of nicotinic acetylcholine receptors (nAChRs) to cholinergic signaling is underscored by the successful development of nicotinic channel agonists as antiparasitics (44–50), but much less is known about the druggability of muscarinic acetylcholine receptors (mAChRs), which are associated with slower but more sustained synaptic and extrasynaptic transmission. The C. elegans genome encodes three known G protein-linked acetylcholine receptors (GARs) (51–54) that are widely expressed in the nervous system and muscle tissues (53, 55) and are involved in the regulation of feeding, mating, egg laying, and locomotion (32, 52, 56–58). While some of the GAR functions in C. elegans are likely conserved in parasitic nematodes, very little is known about GAR biology in filariae and other clade III parasites. GAR-1 from the gastrointestinal nematode Ascaris suum displays atypical pharmacologic responses (25, 59), and muscarinic compounds affect motility in adult-stage B. malayi parasites (60), justifying closer examination of the GAR receptor subfamily.

Here, we focus our efforts on the characterization of a phylum-conserved muscarinic acetylcholine receptor in B. malayi, *Bma*-GAR-3. We examine the effects of muscarinic compounds on microfilariae and adult Brugia parasites using multivariate phenotyping approaches and determine temporal and spatial gene expression patterns for *Bma-gar-3*. Building on previous work (35, 61–64), we exploit the physiological context of C. elegans as a versatile heterologous platform for the study and characterization of parasite GPCRs as anthelmintic targets. We establish functional expression of *Bma*-GAR-3 in C. elegans and tissue-specific phenotypic endpoints in parasitized strains that allow deorphanization (ligand identification) and pharmacologic characterization. Finally, we validate a high-throughput assay that enables screening of *Bma*-GAR-3 expressed in the C. elegans pharynx. These approaches circumvent some of the challenges associated with the study of GPCRs in difficult helminth systems and are likely extensible to many other parasitic nematodes and receptors.

## RESULTS AND DISCUSSION

### Brugia malayi GAR-3 is highly expressed across intramammalian life cycle stages and may mediate whole-organism effects of muscarinic compounds.

**FIG 1:**
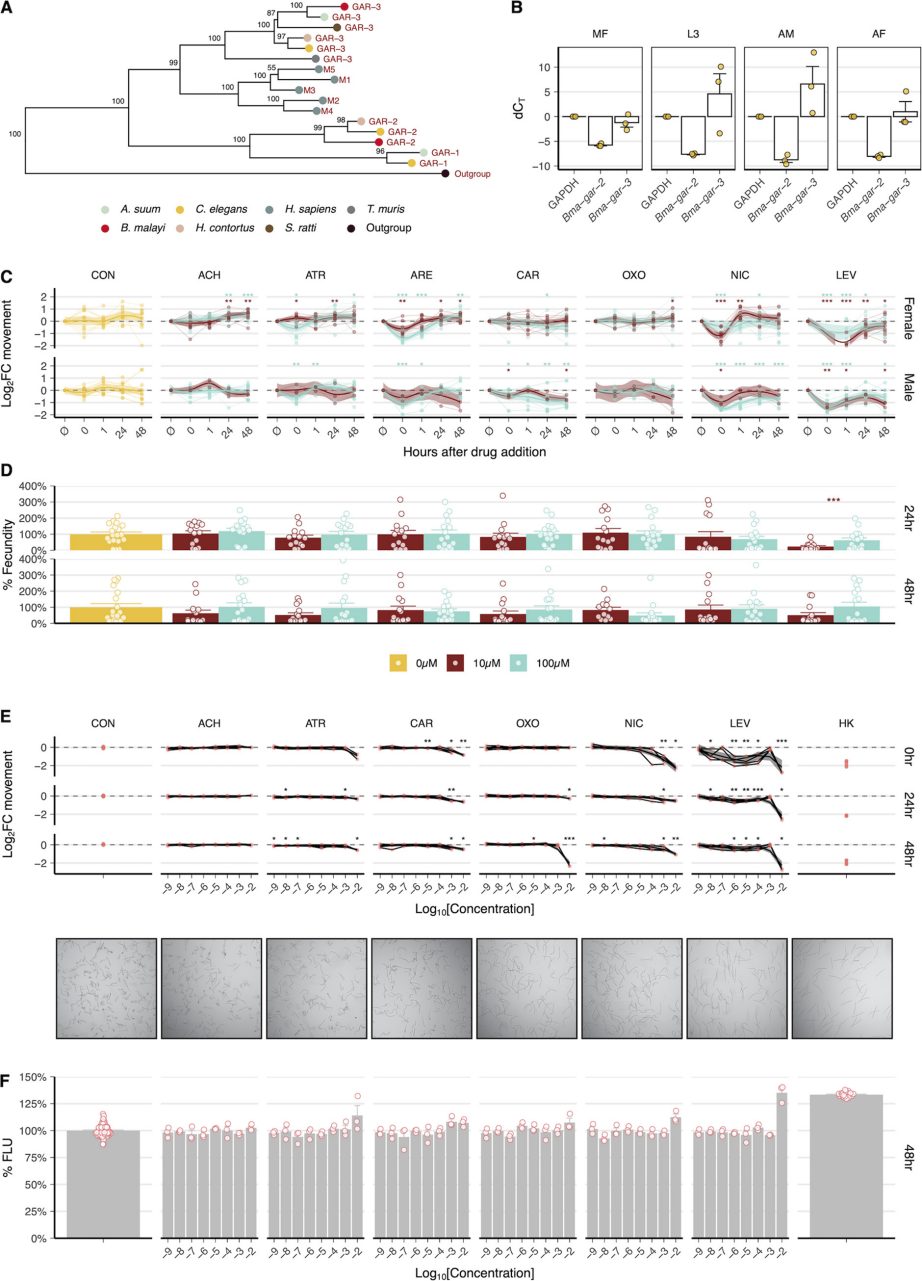
B. malayi expresses two GARs, and muscarinic compounds elicit neuromuscular effects in microfilariae (mf) and adult-stage parasites. (A) Phylogeny based on protein sequence alignment of characterized and putative nematode and human GARs. B. malayi expresses two GARs (*Bma*-GAR-2 and *Bma*-GAR-3) but lacks a GAR-1 homolog. Nodal values represent bootstrap support of 1,000 replicates. (B) Gene expression of *Bma-gar-2* and *Bma-gar-3* across intramammalian life stages of B. malayi as assayed by quantitative reverse transcription-PCR (qRT-PCR) (B. malayi GAPDH is the control). *Bma-gar-3* is abundantly and constitutively expressed throughout the life cycle. (C) Effects of cholinergic compounds on adult B. pahangi movement over 48 h as measured by optical flow. Log_2_ fold change (FC) in normalized movement compared to untreated controls using the *t* test (*, *P* ≤ 0.05; **, *P* ≤ 0.01; ***, *P* ≤ 0.001) (brown, 10 μM; cyan, 100 μM). (D) Effects of cholinergic compounds on adult female B. pahangi fecundity as measured by microfilaria output. Percent changes in mf output compared to untreated controls by time point using the *t* test (*, *P* ≤ 0.05; **, *P* ≤ 0.01; ***, *P* ≤ 0.001) (brown, 10 μM; cyan, 100 μM). (E) Dose-response effects of cholinergic compounds on microfilaria motility over 48 h. Images from mf motility plates taken at 48 h showing diverse morphologies from cholinergic treatment at 10 mM. Log_2_ FC in normalized movement compared to untreated controls using the *t* test (*, *P* ≤ 0.05; **, *P* ≤ 0.01; ***, *P* ≤ 0.001). (F) Effects of cholinergic compounds on microfilaria cell health over 48 h as measured by cell toxicity stain (CellTox fluorescence). Percent changes in fluorescence compared to untreated controls.

Homology-based searches of annotated C. elegans G protein-linked acetylcholine receptors (GARs) were used to identify closely related biogenic amine receptors across six parasitic nematode species. Phylogenetic analysis of putative GARs revealed that B. malayi possessed one-to-one orthologs of C. elegans GAR-2 (*Cel*-GAR-2) and GAR-3, but not GAR-1 (Fig. 1A). The clade IIIb nematode A. suum possesses a GAR-1 ortholog, but GAR-1 could not be identified in the B. malayi clade IIIc sublineage. Although GAR-3 clusters closest to human mAChRs (65), nematode GARs are significantly diverged from their mammalian host orthologs and likely exhibit distinct pharmacological profiles that may allow selective targeting (25, 53, 66). In order to determine the temporal patterns of B. malayi *gar-2* and *gar-3* gene expression, we performed quantitative reverse transcription-PCR (qRT-PCR) across intramammalian life cycle stages (microfilariae [mf], L3, adult male, and adult female). *Bma-gar-3* was constitutively expressed across all life cycle stages and was much more highly expressed than *Bma-gar-2* (Fig. 1B), suggesting potentially outsized physiological roles throughout development.

To explore the gross effects of cholinergic compounds on parasite health, we optimized a number of phenotypic readouts in Brugia adults and microfilariae (67). Parasites were incubated in muscarinic and nicotinic compounds, and the stage-specific effects of these chemical perturbations on worm motility, viability, and fecundity were measured using a customized imaging platform (68). Male and female Brugia adults were exposed to 10 μM and 100 μM acetylcholine (ACh), atropine (ATR), arecholine (ARE), carbachol (CAR), oxotremorine M (OXO), nicotine (NIC), and levamisole (LEV). ACh leads to a slow increase in baseline movement after prolonged exposure (24 and 48 h), attributable to inefficient penetration of the cuticle (Fig. 1C) (69). Treatment with nicotinic compounds (NIC and LEV) leads to an immediate drop in female and male (10 μM and 100 μM) worm motility, followed by a quick recovery mediated by fast-responding nAChRs (70).

Treatment with compounds associated with muscarinic activity (ATR, ARE, CAR, and OXO) elicits a range of subtle-to-large effects on motility. ATR and ARE immediately decrease motility in male and female worms (100 μM) and CAR decreases motility in male worms (10 μM and 100 μM), in agreement with previous work (60). *Cel-*GAR-2 is unaffected by the muscarinic agonists ARE and OXO or the antagonist ATR (53, 65), suggesting that effects driven by these compounds are mediated by *Bma*-GAR-3. Given the promiscuity of some muscarinic compounds, it is possible that some of these effects are partly mediated by nicotinic receptors or that acute effects are dominated by ionotropic as opposed to metabotropic signaling. Fecundity was not significantly altered by any of the treatments except 10 μM LEV at 24 h (Fig. 1D); LEV is a nicotinic compound that stimulates egg laying in C. elegans (71).

Dose-response assays were carried out in mf-stage parasites over three time points (0, 24, and 48 h) to measure the effects of cholinergic compounds on motility and cell death. NIC and LEV both caused immediate inhibition of mf motility, at high (>10^−4^ M) and low (>10^−7^ M) concentrations, respectively (Fig. 1E). OXO, a GAR-3-selective muscarinic compound, significantly decreased motility at high concentrations (>10^−2^ M) in the 24- to 48-h time frame. The morphologies of mf at 48 h varied among treatments, suggesting effects not fully captured by motility. LEV, NIC, and OXO treatment caused worms to become flaccid, much like heat-killed (HK) control worms, while ACh-treated worms maintained the posture of untreated controls. Some cell death was noticeable for all treatments, except acetylcholine, at high concentrations (>1 mM) at 48 h (Fig. 1F). While the OXO-mediated effects suggest that perturbation of *Bma*-GAR-3 may elicit phenotypes of interest, the slower neuromodulatory action of this general receptor class may require assays sensitized to other subtle but important phenotypes in the host context (72, 73). More insight into the tissue-specific expression patterns of this highly-expressed receptor would allow better prediction of its physiological roles.

### *Bma-gar-3* transcripts are widely expressed across critical tissues in adult-stage parasites.

**FIG 2:**
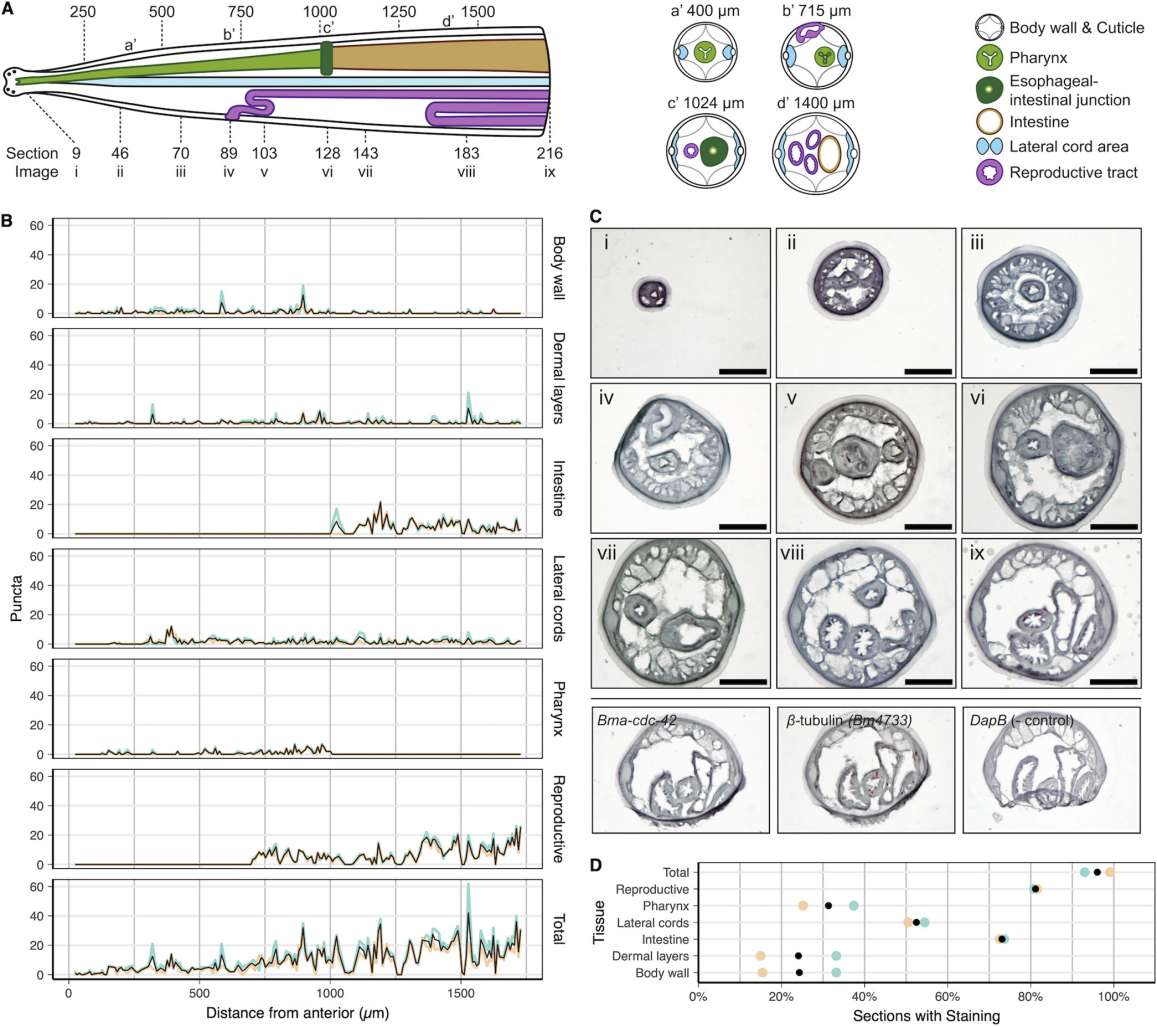
RNAScope spatial localization of *Bma-gar-3* in the B. malayi adult female head. (A) Illustration of tissue distribution along the anterior-posterior axis and transverse illustrations at approximately 400 μm (a’), 715 μm (b’), 1,024 μm (c’), and 1,400 μm (d’), with a key to representative section locations whose images are shown in panel C. (B) RNAScope *Bma-gar-3* puncta counts per tissue sample per 8-μm section. (C) Representative section images as shown by the key in panel A, showing 3× zoom insets of punctate staining in the pharynx (iii), vulva (iv), uterus (v), esophageal-intestinal junction (vi), body wall muscle (vii), intestine (viii), and lateral cords (ix). Scale bar = 50 μm. Positive (*Bma-cdc-42* and *Bm4733*) and negative (bacterial *DapB*) controls for RNAScope validate RNA integrity and show that punctate staining is probe specific and that nonspecific background staining is minimal. (D) Proportions of sections containing *Bma-gar-3* punctae where tissue is present and defined. Colors in panels B and D represent counts performed by two independent researchers.

While GARs have been localized to specific cells and tissues using genetic tools in the tractable C. elegans system, the fine spatial distribution of these receptors is unknown in filarial or other parasitic nematodes. Building on an RNA tomography protocol (74), we developed a strategy to map *Bma-gar-3* transcripts across the adult female head and midsection at 8-μm resolution while preserving spatial information (https://doi.org/10.6084/m9.figshare.20757481.v1). Individual sections were captured sequentially from the anterior tip (216 sections) of a single adult female B. malayi worm, and RNAScope was used to localize *Bma-gar-3* transcripts within sections, allowing the reconstruction of expression patterns down the anterior-posterior axis of the head region (Fig. 2A). *Bma-gar-3* was widely expressed across several tissue types, including the body wall muscle and neurons, with nearly ubiquitous expression in digestive and reproductive tissues (Fig. 2B to D). The expression of *Bma-gar-3* across these important tissues overlaps the expression pattern of C. elegans *gar-3* (body wall muscle, pharyngeal muscle, cord, and other neurons) (75–78), suggesting some conservation of pleiotropic receptor function across clades.

### Heterologous expression and deorphanization of *Bma*-GAR-3 in C. elegans.

We sought to establish heterologous assays to characterize the pharmacology of B. malayi GPCRs through functional expression in discrete C. elegans tissues. Pharmacological profiling of parasite GPCRs in this heterologous system requires that the receptors are properly folded and exported to the membrane, that they signal through endogenous G proteins, and that their activation in response to exogenous ligands can be measured through convenient phenotypic endpoints. Building on previous work leveraging C. elegans as a heterologous expression platform for the expression of human GPCRs (79, 80) and anthelmintic targets (35, 61–63), we first established transgenic lines expressing *Bma-*GAR-3 in the C. elegans ASH sensory amphid neuron and the body wall muscle. These parasitized C. elegans strains were used to develop and optimize tissue-specific assays to measure receptor activation.

**FIG 3:**
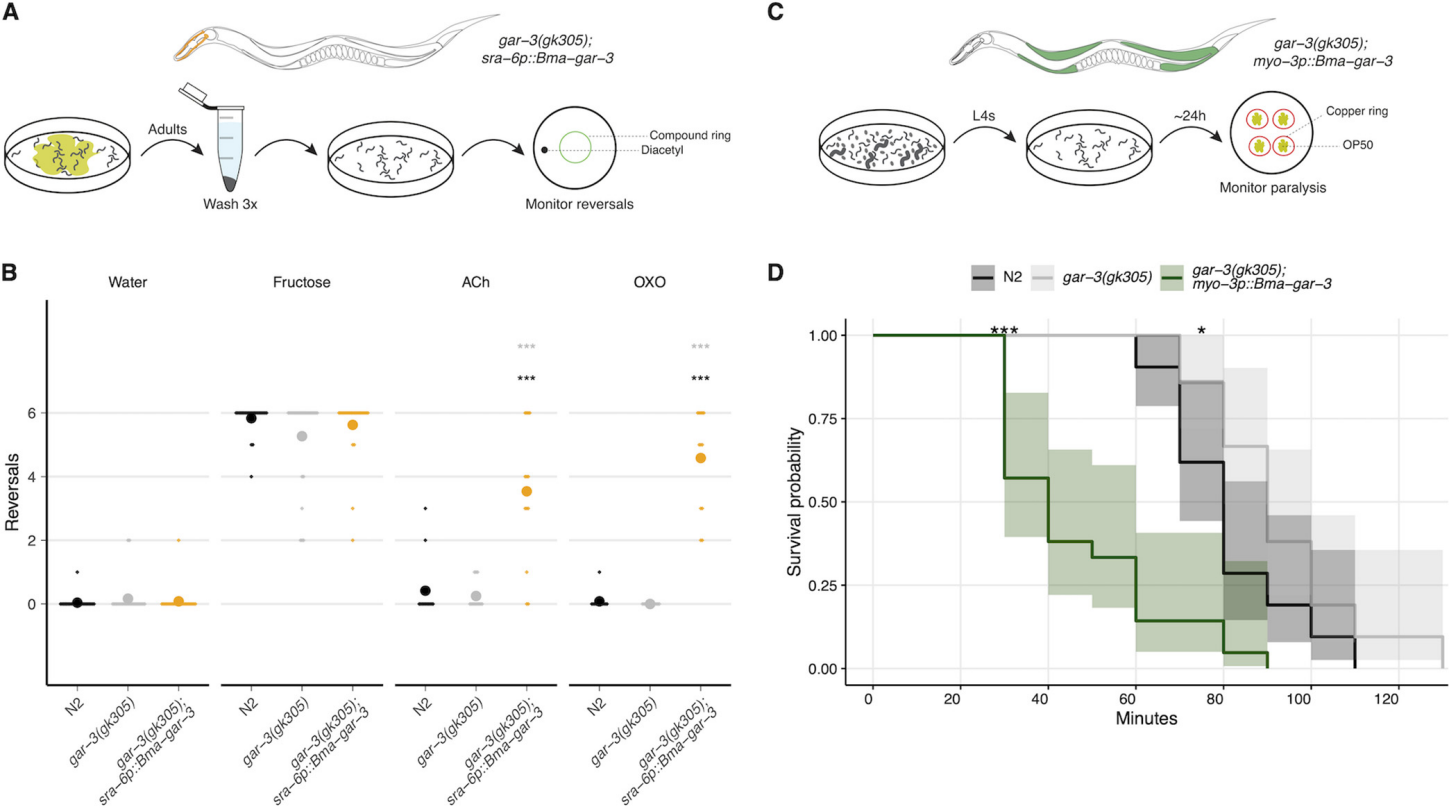
*Bma*-GAR-3 is activated by the selective muscarinic agonist oxotremorine M and confers hypersensitivity to aldicarb-induced paralysis. (A) Schematic of plate-based aversion assay. Bleach-synchronized adult worms are monitored to capture reversal frequency in response to a test compound ring, in the presence of a known attractant (diacetyl). (B) Acetylcholine and oxotremorine M activate the ASH neuron of parasitized C. elegans expressing *Bma*-GAR-3 and elicit reversal behaviors. The *t* test (*, *P* ≤ 0.05; **, *P* ≤ 0.01; ***, *P* ≤ 0.001) was used to identify differences in comparison to *gar-3(gk305)* (gray) and N2 (black). (C) Schematic of aldicarb paralysis assay. Paralysis of young-adult worms was monitored on agar plates containing test drug over a 2-h period. (D) Kaplan-Meier survival plot showing paralysis measured every 10 min. Knockout of *Cel*-GAR-3 leads to resistance to aldicarb-induced paralysis. *myo-3p::Bma-gar-3* worms become paralyzed more rapidly than wild-type N2 and *gar-3(gk305)* worms. Pairwise survival *t* test (*, *P* ≤ 0.05; **, *P* ≤ 0.01; ***, *P* ≤ 0.001).

To limit background signaling, transgenic lines were created in a *Cel-*GAR-3 knockout [*gar-3(gk305)*] genetic background. We employed simple plate-based assays to verify proper cell surface expression of B. malayi GAR-3 and to deorphanize the receptor by confirming activation by the putative ligand ACh. Activation of the C. elegans ASH neuron by noxious stimuli results in a well-characterized avoidance response wherein worms reverse their movement. We hypothesized that the successful activation of parasite GPCRs expressed in this neuron should lead to increased reversal frequency. We adapted an aversion assay (81, 82) that involved placing individual worms in the center of a compound ring and monitoring for reversals in movement in response to test compounds (Fig. 3A). Worms expressing *Bma*-GAR-3 in the ASH neuron (*sra-6p::Bma-gar-3*) exhibited strong aversion responses to ACh (100 mM) and the selective muscarinic agonist oxotremorine M (100 mM) (Fig. 3B). OXO has been shown to specifically activate C. elegans GAR-3, but not *Cel*-GAR-1 or *Cel-*GAR-2 (51, 53, 66). Neither the wild-type (N2) nor knockout [*gar-3(gk305)*] strains demonstrated aversion to ACh or OXO, but all strains maintained consistent responses to negative (water) and positive (4 M fructose) controls.

We then assayed the effects of a cholinesterase inhibitor (aldicarb) on worms expressing *Bma*-GAR-3 in the body wall muscle (*myo-3p::Bma-gar-3*), predicting that the buildup of ACh at the receptor synapse would lead to flaccid paralysis of this parasitized C. elegans strain. We adapted a protocol (83) that involved transferring worms onto plates with 1 mM aldicarb, restricting their movement with copper rings, and monitoring responses to touch stimuli over a 120-min period. *myo-3p::Bma-gar-3* worms were hypersensitive to aldicarb-induced paralysis compared to wild-type and knockout strains (Fig. 3C and D). Combined, these results show that *Bma*-GAR-3 can be functionally expressed in both sensory neurons and body wall muscle and that this receptor is activated by acetylcholine and oxotremorine M.

### Establishing pharyngeal endpoints for profiling *Bma*-GAR-3 pharmacology.

In order to improve the pharmacologic profiling of parasite GPCRs in this heterologous system, we optimized more quantitative assays to measure receptor activation in response to exogenous drugs. Any eventual anthelmintic screen of parasite receptors expressed in this model system will require quantitative and scalable phenotypic readouts of receptor activity. We generated a strain of C. elegans expressing *Bma*-GAR-3 in the pharyngeal muscle (*myo-2p::Bma-gar-3*), hypothesizing that the expression and activation of parasite acetylcholine receptors in the pharynx and the body wall would alter baseline and drug-induced pharyngeal pumping activity. Pharyngeal pumping activity can be directly measured by observing terminal bulb inversions (56, 75, 84) or using electropharyngeogram (EPG) recordings (85, 86). Each pump is highly regulated (57, 87) by muscarinic receptors working in tandem with nAChRs (57, 75, 88).

To optimize assays that rely on pharyngeal function as a quantitative measure of direct or indirect parasite receptor activity, we investigated the effects of pumping stimuli on our ability to resolve drug responses in parasitized strains across worm developmental stages. We first examined how different pharyngeal pumping stimuli would affect our ability to measure drug-induced changes in both larvae and adults. While the food source Escherichia coli strain OP50 and serotonin (5-hydroxytryptamine [5-HT]) are commonly used to elevate baseline pumping frequency (85, 89–93) for assay sensitization, it is important that these stimuli do not mask drug effects and that they allow the reliable capture of both inhibitory and stimulatory responses to drug exposure. We tested combinations of OP50, 5-HT, and a known GAR-3-dependent inhibitor of pharyngeal pumping (arecoline) (75) to assess our ability to capture effects in both directions in L1 and young-adult animals.

**FIG 4:**
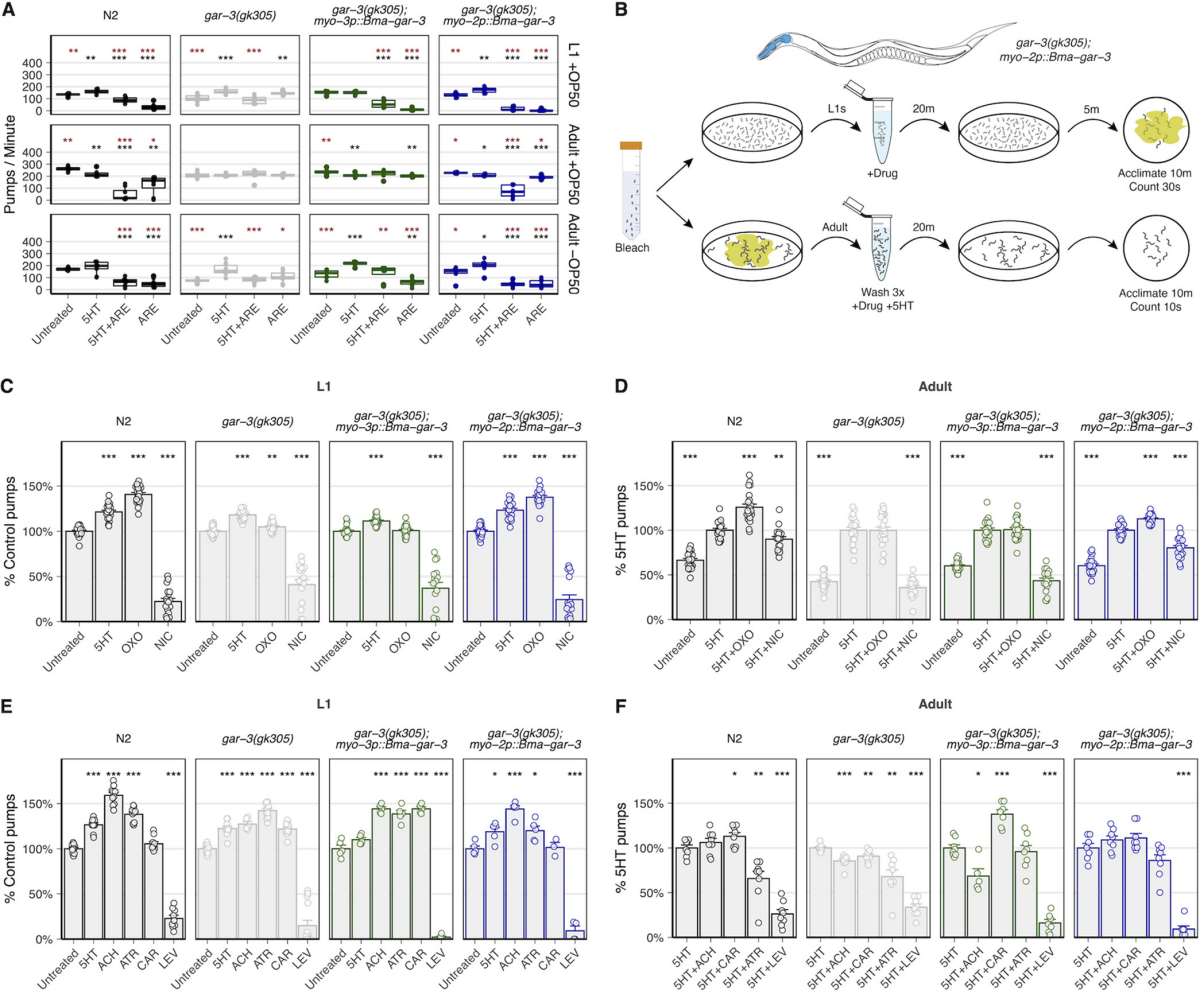
*Bma*-GAR-3 modulates pharyngeal pumping via expression in the C. elegans body wall and pharynx. (A) Iteration across chemical (5-HT) and food (OP50) pumping stimuli for optimization of visual pumping assay in L1- and adult-stage C. elegans worms (brown asterisks, comparisons to 5-HT; black asterisks, comparisons to untreated). (B) Schematic of the optimized visual pharyngeal pumping assay. Bleach-synchronized worms were treated with drug for 20 min, and pumps were counted on seeded (L1s) or unseeded (adults) 6-cm agar plates. Drug treatments were tested in combination with 5-HT in the adult stage. (C and D) Modulation of pharyngeal pumping by OXO in L1 and adult parasitized strains. L1 data were normalized to untreated controls, while adult data were normalized to 5-HT-stimulated controls. (E and F) Effects of cholinergic compounds on stage-specific pharyngeal pump frequency compared to untreated (L1) or 5-HT-stimulated (adult) pump frequency. All statistics were calculated using the *t* test (*, *P* ≤ 0.05; **, *P* ≤ 0.01; ***, *P* ≤ 0.001).

L1 assays carried out on OP50 plates confirmed that this food stimulant did not saturate pumping responses or mask expected drug effects. The inhibitory effect of arecoline could be measured in strains expressing either C. elegans GAR-3 (N2) or B. malayi GAR-3 (*myo-2p::Bma-gar-3* strain) in the pharynx (Fig. 4A). A dynamic range of pump frequencies was observable using OP50, and we concluded from these data that L1 assays should be carried out in the presence of OP50 and without 5-HT. OP50 led to a nonsaturating increase in L1 baseline pharyngeal pumping that allowed us to measure both drug-induced stimulation and inhibition of pump frequency. In contrast, adult-stage assays carried out in the presence of OP50 saturated baseline pharyngeal pumping frequency. The addition of 5-HT led to no further increase in pumping rate, and the inhibitory effects of ARE were largely masked in these conditions (Fig. 4A). Assays carried out in the absence of OP50 allowed the robust detection of both 5-HT stimulation and arecoline inhibition. We concluded from these data that adult assays should be carried out in the absence of OP50 and with drugs in combination with 5-HT to allow the largest dynamic range of stimulation and inhibition.

We next used these optimized L1 and adult-stage assays (Fig. 4B) to confirm the action of the selective *Cel-*GAR-3 agonist oxotremorine M. OXO increased the pumping frequency in L1-stage (~41%) and adult-stage (~26%) N2 worms (Fig. 4C and D). Knockout of native *gar-3* led to a loss of OXO responsiveness, which was nearly completely rescued by the expression of *Bma-gar-3* in the pharynx but not the body wall muscle. We next profiled the responses of parasitized strains to muscarinic and nicotinic compounds with less receptor specificity. In L1-stage worms, the expression of *Bma-gar-3* in the pharynx restored the wild-type response profile (Fig. 4E). In adult-stage worms, the expression of *Bma-gar-3* in the body wall led to hyperstimulation of pumping in response to CAR, while the expression of *Bma-gar-3* in both the pharynx and body wall led to a decreased inhibitory response to ATR compared with that of either the wild-type or the *gar-3(gk305)* strain (Fig. 4F).

Although it is known that pharyngeal pumping can be modulated by cholinergic signaling in both tissue types, the precise mechanism by which the body wall and pharynx communicate is unclear (86, 94, 95). Interpretations of how pharyngeal pumping is modulated by direct versus indirect pharmacological action at our receptor of interest can be confounded by the promiscuous binding of cholinergic compounds to a range of muscarinic and nicotinic receptors expressed across relevant tissues and perhaps differentially expressed across stages. Despite these complications, it is reasonable to expect that compounds with high specificity for GAR-3 can be identified by comparing responses across *gar-3(gk305)* and *gar-3(gk305); myo-2p::Bma-gar-3* strains.

### Electropharyngeal measurements of *Bma*-GAR-3 activity in parasitized strains.

**FIG 5:**
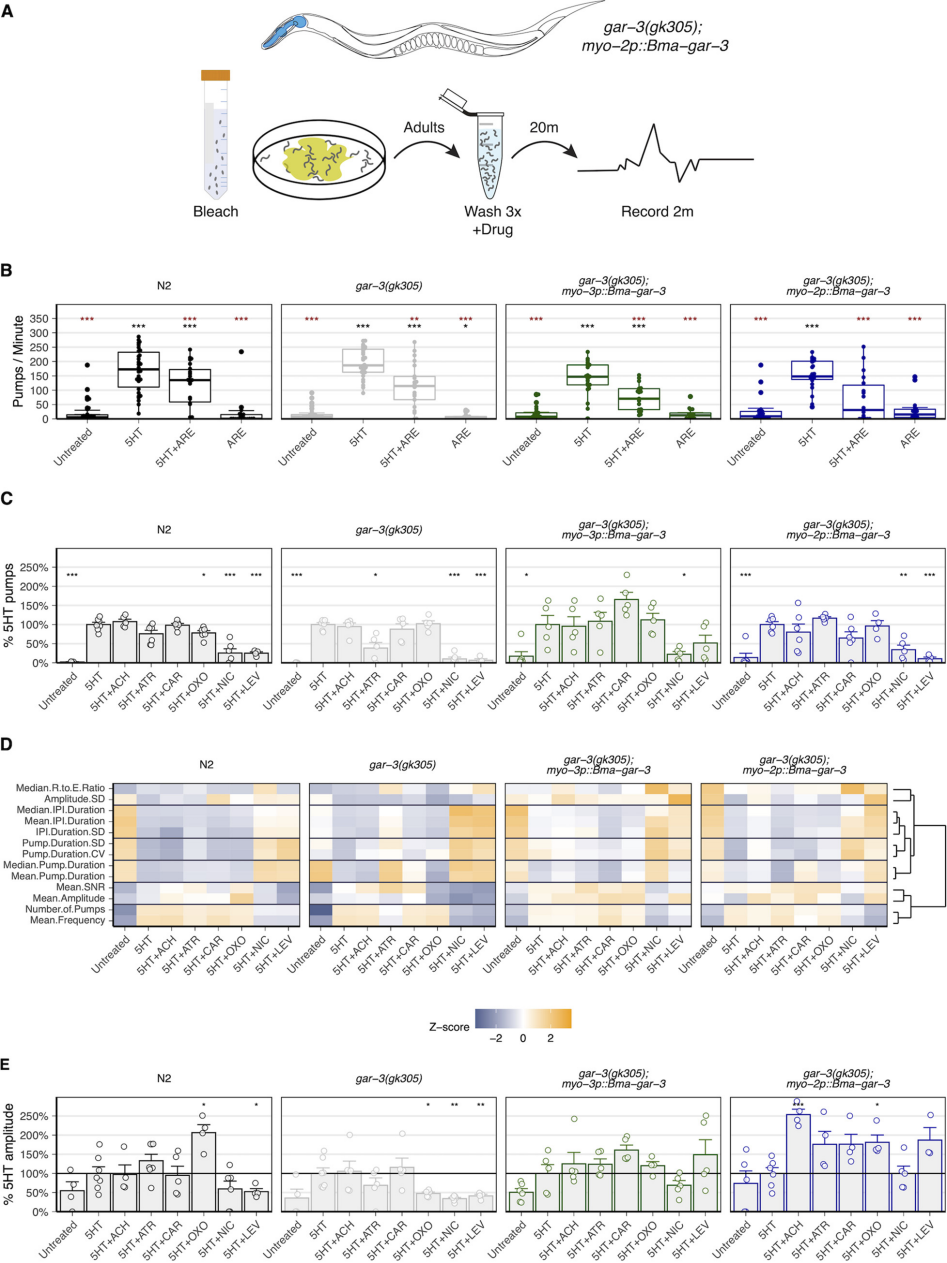
EPG recordings in parasitized strains provide alternative reporters of parasite receptor activity. (A) Schematic of electropharyngeogram (EPG) assay. Adult worms were bleach synchronized, washed three times, treated with drugs for 20 min, and positioned into the microfluidics chip. Worms were recorded for 2 min. (B) EPG recordings used to measure pharyngeal pump frequency show that 5-HT in combination with drugs allows the capture of inhibitory effects of ARE. (brown asterisks, comparisons to 5-HT; black asterisks, comparisons to untreated). (C) Cholinergic effects on pharyngeal pump frequency as measured by EPG do not align with visual pumping assay. (D) Heatmap depicting scale-normalized electrophysiological features across all strain conditions. Clustering of these features identifies subsets that provide similar information. (E) *Bma*-GAR-3 expressed in the pharynx increases the peak amplitude of OXO-treated worms compared to *gar-3(gk305)* knockout worms, revealing receptor-specific modulation of this electrophysiological feature. All statistics were calculated using the *t* test (*, *P* ≤ 0.05; **, *P* ≤ 0.01; ***, *P* ≤ 0.001).

Electrophysiological recordings from the pharynx can provide more detailed information about pharyngeal function in response to a drug. Using established protocols (85), we sought to use electropharyngeogram (EPG) recordings to investigate other pharyngeal phenotypes modulated by *Bma-*GAR-3 expression in the body wall and pharynx of young-adult C. elegans worms. We recorded individual worms for 2 min after a 20-min drug incubation period, mirroring the visual counting assay (Fig. 5A). The use of 5-HT in combination with test drugs was necessary to capture the inhibitory effects of ARE and to recapitulate trends from visual counting data (Fig. 5B).

We next used EPG recordings to test whether the effects of cholinergics on electrophysiological features could be linked to *Bma*-GAR-3 activity in parasitized strains. We found that EPG-derived pump frequency did not correlate well with visual counting data. Most notably, OXO did not exhibit a pattern of differential response and rescue in *gar-3(gk305)* and *gar-3(gk305); myo-2p::Bma-gar-3* worms, respectively (Fig. 5C). General discrepancies between visual counts and EPG-derived pump frequency are likely due to the disconnect between terminal bulb movement and action potentials, supported by the fact that C. elegans GAR-3 regulates both membrane potential and excitation-contraction coupling through an unresolved signaling pathway (75). While EPG-derived pump frequency was not a reporter of *Bma-*GAR-3 activation in this assay, other electrophysiologic features showed patterns consistent with *Bma-*GAR-3 phenotypic rescue (Fig. 5D). Specifically, the expression of *Bma-gar-3* in the pharynx rescued the OXO-induced increase in peak amplitude that was lost in the *gar-3(gk305)* background (Fig. 5E). The EPG recordings provided a rich set of features (Fig. S1 in the supplemental material) that can reveal the activation of parasite receptors in response to exogenous drug. While these electrophysiological assays provide deeper insight into electric and chemical signaling dynamics, they do not enable high-throughput screening (HTS) of parasitized strains to identify drugs that act on receptors of interest.

### Establishing a high-throughput image-based feeding assay for screening of parasitized strains.

We developed a high-throughput imaging assay to measure pharyngeal pumping as a reporter of receptor activity in parasitized animals. Fluorescence uptake in the form of beads, bacteria, and dye has been used to measure feeding behaviors in C. elegans, whereby drug modulation of pumping rates can be expected to impact the amount of intestinal fluorescence. Many of these assays are low in throughput (96–99) or require a large particle sorter (100–103) or luminometer (84), necessitating the development of a high-throughput and high-content imaging endpoint that allows the measurement of intestinal fluorescence and transgenic markers.

**FIG 6:**
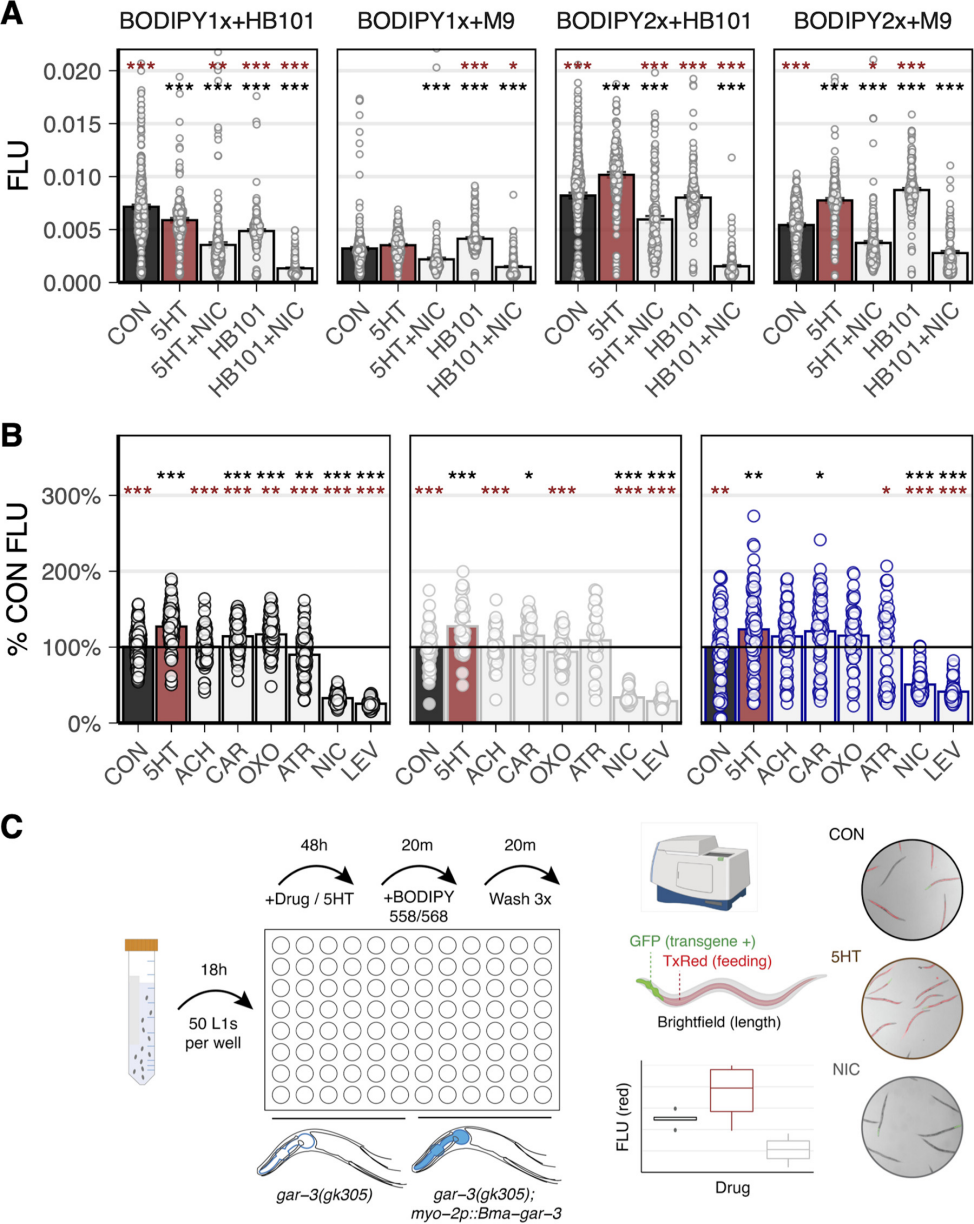
Development of an image-based feeding assay to enable high-throughput screening of parasitized strains. (A) Testing lipophilic dye (BODIPY) concentration (1×, 45 ng/mL; 2×: 90 ng/mL) and the inclusion of E. coli strain HB101 for the development of a feeding assay. Treatment with 2× BODIPY in the absence of HB101 allowed the detection of both pharyngeal stimulation and inhibition as quantified by the accumulation of red intestinal fluorescence. *t* test (*, *P* ≤ 0.05; **, *P* ≤ 0.01; ***, *P* ≤ 0.001; brown, comparisons to 5-HT; black, comparisons to untreated). (B) Feeding assay carried out using a panel of cholinergic treatments. *Bma*-GAR-3 expression in the pharynx partially rescues the wild-type OXO effect that is lost in the *gar-3(gk305)* background, as measured by fluorescent dye uptake. Strains: N2 (left), *gar-3(gk305)* (center), and *gar-3(gk305); myo-2p::Bma-gar-3* (right). *t* test (*, *P* ≤ 0.05; **, *P* ≤ 0.01; ***, *P* ≤ 0.001; brown, comparisons to 5-HT; black, comparisons to untreated). (C) Schematic of the dye feeding assay. Bleach-synchronized L1 worms were grown in 96-well plates for 48 h, followed by a 20-min drug treatment. Worms were then fed >90 ng/mL BODIPY 558/568 for 20 min. Plates were washed three times, and worms were paralyzed and straightened with 50 mM sodium azide before images were acquired using a high-content imaging system. Fluorescence was quantified using a wrmXpress (68) pipeline. Created in part with BioRender.com.

We optimized the parameters for a microtiter-plate assay that measures intestinal accumulation of fluorescent dye as a correlate of pharyngeal activity. L1-synchronized worms were aliquoted into 96-well plates and grown to adults over 48 h in the presence of E. coli strain HB101. The worms were then treated with a test compound for 20 min, followed by a 20-min BODIPY 558/568 (red) incubation. We tested two concentrations of BODIPY to help minimize background fluorescence and ensure that the dye was not a limiting reagent. We tested the inclusion of 5-HT and HB101 as feeding stimulants for the duration of the drug exposure, as well as the inclusion of HB101 in the BODIPY incubation period. Assays using at least 90 ng/mL BODIPY (2×) generated the best conditions to detect both pharyngeal stimulation (5-HT and HB101) and inhibition (NIC) (Fig. 6A). The inclusion of HB101 in the dye incubation period was not necessary to detect these differences (Fig. 6A).

To validate this protocol as a means to screen parasite receptors at higher throughput, we compared the effects of cholinergic compounds in *gar-3(gk305)* and *gar-3(gk305); myo-2p::Bma-gar-3* animals. We treated worms with cholinergic compounds, followed by BODIPY incubation. An image-processing pipeline was established to identify transgene (+) worms via pharyngeal GFP expression. OXO caused an increase in dye uptake in wild-type worms compared to the uptake in nontreated controls (~17%, *P* = 9.4e−07), consistent with the increased pumping frequency observed in visual counting assays. OXO decreased dye uptake in the *gar-3(gk305)* background, which was rescued by the expression of *Bma-gar-3* in the pharynx (Fig. 6B). These results reaffirm that OXO has selective effects on *Bma-*GAR-3 in the pharynx and provide proof of principle for this high-content imaging approach for pharmacological profiling of transgenically expressed parasite GPCRs.

### Conclusion.

Parasite G protein-coupled receptors remain unexploited as anthelmintic targets despite their involvement in critical nematode neuromuscular and physiological processes. One significant bottleneck in exploring the pharmacology of parasite GPCRs results from difficulties in consistently establishing heterologous expression in single-cell systems. Yeast and mammalian cell culture systems have paved the way for deorphanization of helminth GPCRs (18, 25, 104), but not all receptors express or behave properly in cell types derived from distant phylogenetic lineages. The combinations of accessory proteins, molecular chaperones, G proteins, and membrane determinants required for the successful folding, cell surface expression, and signaling of parasite receptors in surrogate systems have not been comprehensively identified. To avoid some of these complications, we explored a range of assays for parasite GPCR expression and profiling in the model nematode C. elegans.

We identified a B. malayi muscarinic GPCR (*Bma-gar-3*) that was highly expressed throughout the intramammalian life stages and developed a spatial RNAScope protocol to map receptor transcripts in multiple tissue types in the adult stage. Multivariate phenotypic assays of cholinergic effects on microfilariae and adult parasites revealed differential effects of nicotinic and muscarinic agents. We showed that muscarinic compounds affected motility in both adult- and mf-stage parasites, some of which were likely to be mediated by GAR-3. We predict that sampling a broader array of muscarinic compounds will likely reveal other overt and subtle-but-important phenotypes that are relevant to potential anthelmintic mechanisms of action.

Building upon previous work (35, 61–64), we expressed *Bma-gar-3* in the C. elegans body wall muscle, pharynx, and sensory neurons. Different phenotypic endpoints were optimized to measure receptor activity across these parasitized strains. Simple plate-based assays allowed the deorphanization of *Bma*-GAR-3 expressed in the body wall and sensory neurons. We focused primarily on pharyngeal expression, given its amenability to a range of visual, electrophysiological, and imaging assays. While visual observations of pharyngeal pumping and electropharyngeogram recordings provided different measurements of *Bma*-GAR-3 perturbation, these assays were ultimately low in throughput. To enable more facile and higher-throughput screening of pharynx-expressed receptors, we deployed a microtiter-plate imaging assay that measured the accumulation of lipophilic dye as a reporter of pharyngeal pumping. We show that this feeding assay can be used to detect activation of *Bma*-GAR-3 within the pharynx.

The suitability of these approaches for a given parasite GPCR will depend on the complement of related receptors and endogenous ligands that signal in targeted tissues. We show that transgenic strains can be created in various genetic knockout backgrounds to help mitigate some of these concerns. While expression in scalable single-cell systems will remain an important objective for high-throughput screening (HTS) against GPCR targets, functional parasite receptor assays in a more native nematode cell and physiological environment can provide important baseline pharmacological data and transgenic whole-organism assays can conceivably be adapted for high-throughput screening and anthelmintic discovery.

## MATERIALS AND METHODS

### Parasites and chemical reagents.

B. malayi and Brugia pahangi adults extracted from the Meriones unguiculatus infection system (NIH/NIAID Filariasis Research Reagent Resource Center) (105) were maintained in daily changes of RPMI 1640 medium with l-glutamine (Sigma-Aldrich) supplemented with fetal bovine serum (FBS) (10%, vol/vol; Fisher Scientific) and penicillin-streptomycin (0.1 mg/mL; Gibco) at 37°C with 5% CO_2_ unless otherwise specified. Brugia microfilariae isolated from the same system were maintained in RPMI 1640 medium supplemented with l-glutamine (Sigma-Aldrich) and penicillin-streptomycin (0.1 mg/mL; Gibco) at 37°C with 5% CO_2_ unless otherwise specified.

The chemicals used in the assays included serotonin (catalog number AAB2126306; Fisher Scientific), arecoline (catalog number AC250130050; Fisher Scientific), atropine (catalog number sc-252392; Santa Cruz Biotechnology), nicotine (catalog number sc-482740; Santa Cruz Biotechnology), levamisole (catalog number TCL0231-1G; VWR), carbachol (catalog number sc-202092; Santa Cruz Biotechnology), acetylcholine (catalog number AC159170050; Fisher Scientific), oxotremorine M (catalog number sc-203656; Santa Cruz Biotechnology), and aldicarb (catalog number sc-254939; Santa Cruz Biotechnology).

### Phylogenetics.

Putative parasite GARs were identified using a reciprocal BLASTp (106) approach using known C. elegans GARs. This initial list of GARs was expanded with homology-based searches against the C. elegans predicted proteome, and a broader list of C. elegans (107) biogenic amine receptors was used to carry out BLASTp searches against the predicted proteomes of B. malayi (108), Ancylostoma caninum (109), Ascaris suum (110), Haemonchus contortus (111), Strongyloides ratti (112), and Trichuris muris (113) (WormBase ParaSite version 16 [114]). Filtered hits (percent identity, >30%; E value, <10^−4^; and percent coverage, >40%) that survived a reciprocal BLASTp search against C. elegans were retained. This list was combined with human muscarinic receptors for phylogenetic inference and annotation. Receptors were aligned with MAFFT (115) and trimmed with trimAl (116), and phylogenetic trees were inferred with IQ-TREE (117). Bootstrap values from 1,000 replicates were drawn as nodal support onto the maximum-likelihood tree. Trees were visualized and annotated with ggtree (118).

### Adult parasite assays.

Multivariate phenotyping of adult parasites was performed as described previously (67, 119). After receipt from the FR3, adult male and female B. pahangi parasites were manually sorted into 24-well plates filled with 750 μL of complete medium (RPMI 1640 plus 10% FBS and penicillin/streptomycin) per well. The parasites were incubated overnight, after which individual parasites were transferred to new plates with 750 μL incomplete medium (RPMI 1640 plus penicillin/streptomycin). Compound stocks (100×) were made fresh daily in H_2_O. Plates were recorded for 15 s, compound was added, and plates were immediately recorded again. Recordings were taken 1 h posttreatment and 24 h posttreatment, prior to transferring parasites to a new preloaded drug plate. The final recordings were taken 48 h posttreatment. Three biological replicates from separate batches of parasite infection cohorts were assayed with four worms per treatment. Videos were analyzed with the optical flow (motility) module of wrmXpress (68).

Conditioned medium from female worms from the initial overnight incubation in complete medium and 24-/48-h treatment plates was transferred to 1.5-mL tubes and centrifuged for 5 min at 10,000 × *g* to pellet progeny. Five hundred microliters of the supernatant was removed, and the remainder was stored at 4°C for >48 h. To quantify progeny, 50-μL aliquots were transferred to wells of a 96-well plate (Greiner), and each well was imaged with transmitted light at 2× with an ImageXpress Nano system (Molecular Devices). Images of progeny were segmented as previously described (67), and segmented pixels were counted to infer output of progeny using a previously generated model (67).

### Microfilaria assays.

B. malayi microfilaria motility and cell toxicity assays were performed as described previously (67, 120). Briefly, mf were purified into culture medium using a PD-10 desalting column (catalog number 95017-001; VWR) and the titers determined to a concentration of 10 mf/μL. An amount of 100 μL containing 1,000 mf was added to each well of a 96-well plate. Heat-killed controls were incubated for 1 h at 60°C before being added to wells. Serial dilutions of 100 mM stock of each drug were freshly made in water. Plates were imaged immediately after treatment (0 h) and 24 and 48 h after treatment. At least two replicates with high technical replication were run for motility assays. CellTox (catalog number G8742; Promega) staining was performed at 48 h, following the kit protocol except with half the recommended concentration of CellTox. Wells were imaged using an ImageXpress Nano (Molecular Devices). Three replicate dose-response plates were assayed using CellTox assays. Videos were analyzed with the motility and segmentation modules of wrmXpress (68), and output data was analyzed using the R statistical software.

### qRT-PCR.

Parasites were flash frozen in liquid N_2_ in 1.5-mL tubes and stored at −80°C in TRIzol LS (catalog number 10296028; Thermo Scientific) in batches of 500,000 (mf), 300 to 500 (L3), or 3 (adults). Three independent samples originating from different batches of animal infections were collected for each stage (mf, L3, adult male, and adult female). Freeze-thawed samples were homogenized using a compact bead mill (TissueLyser LT; Invitrogen), and RNA was extracted using the Zymo Direct-zol RNA miniprep kit (catalog number R2050). RNA integrity and concentration were assessed via NanoDrop, and cDNA was generated with the SuperScript III kit (catalog number 18080051; Thermo Fisher) using equal amounts of oligo(dT) and random hexamer primers for first-strand synthesis. qPCR primers for *Bma-gar-2* (F, 5′-TAATACGACTCACTATAGGGCGACGTACTTCCTCCGATGT-3′, and R, 5′-TAATACGACTCACTATAGGGCCGCTCATCGTATTCCATTT-3′) and *Bma-gar-3* (F, 5′-TTTGGCCACCATGGATTATT-3′, and R, 5′-TGTATAACGCAACGGTCAGG-3′) were designed with Primer3 (121) and optimized to quantify expression levels from cDNA. Glyceraldehyde-3-phosphate dehydrogenase (GAPDH) primers (122) were used as a control. Quantitative reverse transcription-PCRs (qRT-PCRs) in 20-μL reaction mixture volumes were carried out using 2× PowerUp SYBR green master mix (catalog number A25776; Fisher Scientific), 800 nM primers, and 10 ng of cDNA as the input. Reactions were run in triplicate on a StepOnePlus real-time PCR system with the following program: 2 min for 50°C, 95°C for 5 min, 40 cycles of 95°C for 15 s, 55°C for 15 s, and 72°C for 1 min. Cycle threshold (*C_T_*) values were calculated with the system’s automatic threshold.

### RNAScope *in situ* hybridization.

Adult B. malayi females were cultured overnight and separated in 10-cm petri dishes, dipped in 70% ethanol, and spatially embedded in 1% Bacto-agar. The blocks of Bacto-agar were dehydrated for 5 min in 25%, 50%, and 70% ethanol sequentially and stored in 100% ethanol overnight. The Bacto-agar-embedded tissue was processed into a paraffin block, and agar was trimmed from the anterior tip of the agar block before embedding for cross-sectioning. Blocks were sectioned at 8 μm, and sections were arranged sequentially on slides, keeping all sections.

The hybridization probes used for the RNAScope assays (ACD Bio) included DapB (dihydrodipicolinate reductase, Bacillus subtilis) as a negative control and two highly-expressed B. malayi genes as positive controls: *Bma-Bm4733* (B. malayi β-tubulin; positions 2 to 1031 of the sequence with accession number XM_001896580.2 were targeted [20ZZ]) and *Bma*-*cdc-42* (B. malayi putative GTP-binding protein; positions 2 to 550 of the sequence with accession number XM_001899971.1 were targeted [11ZZ]). Our *Bma-*GAR-3 target probe targeted positions 400 to 1501 of the sequence with accession number XM_043081323.1 (20ZZ). The standard RNAScope 2.5 HD assay-red kit (ACD Bio) protocol was followed with an adjusted length of 8 min for retrieval using a steamer and 45 min for amp 5. Imaging was done in bright-field microscopy with a 40× Nikon Plan Apo objective on a Nikon Eclipse80i. The presence of red punctate staining, indicating target gene expression, was quantified by manual annotation by two independent observers. Annotation and alignment were carried out with Fiji (123) using TrakEM2 (124).

### Cloning and transgenic C. elegans strains.

The open reading frame (ORF) for the longest predicted isoform of *Bma-gar-3* (isoform a; WormBase ParaSite version 17) was selected for study based on manual assessment and alignment with homologous GARs. This isoform is supported by long-read RNA-sequencing data (125) that extend the model upstream from the 5′ end of isoform b. The ORF was synthesized (GenScript) and cloned into pPD133.48, L4663 (a gift from Andrew Fire, Addgene plasmid #1665), using BamHI/KpnI sites, to create pMZ0005 (*myo3p::Bma-gar-3::unc-54-3′UTR*). pMZ0012 (*sra-6p::Bma-gar-3::unc-54-3′UTR*) was created by subcloning *Bma-gar-3* into sra-6p::ChR2*YFP (a gift from Shawn Lockery [126]) using BamHI/EcoRI sites. pMZ0018 (*myo-2p::Bma-gar-3::unc-54-3′UTR*) was created by amplifying *myo-2p* from pPD96.48, L2531 (a gift from Andrew Fire, Addgene plasmid #1607), with 5′ XbaI and 3′ BamHI sites and using this amplicon to replace *myo-3p* in pMZ0012.

The genotypes generated and used in this study include ZAM7 (*gar-3(gk305)* V, *maz7Ex*[*sra-6p::Bma-gar-3::unc-54 3′UTR; sra-6p::GCaMP3; unc-122p::GFP*]), ZAM10 (*gar-3(gk305)* V, *maz10Ex*[*myo-3p::Bma-gar-3::unc-54 3′UTR; myo-2p::GFP*]), and ZAM11 (*gar-3(gk305)* V, *maz11Ex*[*myo-2p::Bma-gar-3::unc-54 3′UTR; myo-2p::GFP*]), created as described previously (127) by injecting pMZ0012 (75 ng/μL), pMZ0005 (30 ng/μL), and pMZ0018 (30 ng/μL), respectively, into *gar-3(gk305)* along with fluorescent markers (*unc-122p::GFP* or *myo-2p::GFP*) and empty vector (pPD95.75) to create a final concentration of 100 ng/μL. ZAM19 ([*myo-2p::GFP*]) and ZAM20 (*gar-3(gk305)* V [*myo-2p::GFP*]) were created by injecting 10 ng/μL of a fluorescent marker (*myo-2p::GFP*) and empty vector (pPD95.75) to create a final concentration of 100 ng/μL. VC657(*gar-3(gk305)* V) was sourced from the C. elegans Gene Knockout Consortium (128). Lines were maintained at 20°C on nematode growth medium (NGM) plates seeded with E. coli strain OP50 and routinely picked to fresh plates at the L4 stage.

### Ring aversion assay.

Bleach-synchronized adult worms were rinsed three times in M9 buffer (3 g KH_2_PO_4_, 6 g Na_2_HPO_4_, 5 g NaCl, 1 ml 1 M MgSO_4_, H_2_O to 1 liter) and pipetted onto unseeded 6-cm plates. To assemble the assay plate, a copper ring (catalog number 17668; PlumbMaster) was soaked in a test compound with fast green dye as a visual marker and placed in the center of the plate. One microliter of 1:1,000-diluted diacetyl (attractant) was combined with 1 μL fast green and added outside the ring. Plates were used immediately after assembly. The copper ring was removed after 1 min to allow the test compound to soak into the agar. Individual worms were picked without bacteria to the center of the compound ring on assay plates and monitored for reversals in response to test compounds. Three biological replicates from three independent bleaches were run using four worms per treatment per strain. Observations were made until either the worm crossed the compound ring or six attempts to cross the compound ring occurred without the worm crossing. Water was used as a negative control, and 4 M d-fructose was used as a positive control.

### Aldicarb paralysis assay.

Plates with 1 mM aldicarb were made by diluting 100 mM aldicarb (in 70% ethanol) in NGM. Plates were stored at 4°C and used within 30 days. Thirty L4-stage animals from each strain were picked to OP50-seeded plates and cultured at 20°C overnight until they reached the young-adult stage. Aldicarb plates were acclimated to room temperature on the morning of the assay. Four copper rings (catalog number 17668; PlumbMaster) were dipped in 70% ethanol and briefly flamed before placement onto a single aldicarb plate to form quadrants. Ten microliters of OP50 from an overnight liquid culture was spotted into the center of each of the copper rings and allowed to dry for 30 min. A minimum of 10 worms per strain were picked to the center of a quadrant, allowing the testing of four strains per plate. Four biological replicates were carried out across strains. After 30 min and every 10 min thereafter, each worm was tapped 3 times on the head and tail, the paralyzed worms were removed, and the remaining worms were tallied. This process continued until the 2-h mark.

### Visual pharyngeal pumping assay.

For L1-stage assays, gravid worms were bleached and embryos were hatched on unseeded 10-cm NGM plates overnight (2,000 embryos per plate). L1s were washed from plates into 1.5-mL tubes and treated with drug for 20 min. Five hundred microliters of the treated worm mixture was then transferred to an unseeded 10-cm NGM plate and allowed to dry for 5 min. Individual transgene (+) worms were then picked to seeded 6-cm NGM assay plates and given 10 min to acclimate. Adult assays required minor modifications of the L1 protocol. Young adults were developed on seeded 10-cm NGM plates, drug-treated worms were transferred to 10-cm plates with no drying period, and transgene (+) worms were picked to unseeded 6-cm NGM assay plates. For optimization and cholinergic assays, an average of 8 worms were visually phenotyped for each strain-condition combination. Pumps were measured by the motion of the terminal bulb grinder over a 30-s (L1s) or 10-s (adults) interval using a Zeiss Axio Vert.A1 at ×10 magnification with differential interference contrast (DIC) optics.

### EPG recordings.

Electropharyngeograms (EPGs) were recorded with the ScreenChip system (InVivo Biosystems). Briefly, worms were bleach synchronized and embryos were hatched and developed on seeded 10-cm NGM plates. Young-adult worms were washed 3 times with M9 in 1.5-mL tubes. Worms were treated for 20 min before loading them into the ScreenChip40 microfluidic chamber. Each worm was vacuumed into the channel, acclimated for 30 s, and recorded for 2 min using the NemaAcquire 2.1 software. Worms from three biological replicates representing independent bleaches were assayed for optimization (minimum of 15 worms per condition), and one replicate was assayed for the cholinergic panel (minimum of 5 worms per condition). Analysis was done using NemaAnalysis 0.2 with default settings found under customize analysis. Filtering of data was done by modifying “parameters left” for signal-to-noise ratio (SNR), E and R high-pass cutoffs, and the minimum absolute threshold, followed by visually ensuring all pumps were identified and background noise was ignored.

### Image-based feeding assay.

Bleach-synchronized larval-stage worms (L1) were aliquoted into 96-well plates at a titer of 50 L1 animals per well along with E. coli HB101 bacterial food (2.5 mg/mL final concentration). Worms were incubated for 48 h at 20°C with shaking at 180 RPM until reaching adult stage (129). For the initial screen, adult-stage worms were drug treated for 20 min, followed by the addition of 180 ng/mL BODIPY 558/568 (catalog number D3835; Thermo Scientific) combined with HB101, and again incubated for 20 min. For optimization assays, 45 ng/mL (1×) or 90 ng/mL (2×) BODIPY 558/568 combined with M9 or HB101 was added to the plate with the same incubation time. Plates were washed 3 times with M9, and worms paralyzed with 50 mM sodium azide. Images of worms in transmitted light, green fluorescent protein (GFP) fluorescence, and TxRed fluorescence were taken on an ImageXpress Nano (Molecular Devices) at ×2 magnification. Images were analyzed with the feeding module of wrmXpress, which incorporates a custom CellProfiler model using the Worm Toolbox plugin (130, 131). Segmented worms were computationally straightened, and GFP and TxRed signals were quantified. Transgenic worms were identified using a robust cutoff of GFP quantification such that only worms expressing *Bma-gar-3* were analyzed (Fig. S2A and B). Nonworm objects and contaminating fluorescence were further filtered with size thresholds and outlier pruning (TxRed fluorescence units [FLU] Z score of >3). Total fluorescent dye uptake for GFP-positive worms was quantified using the StdIntensity parameter.

### Data availability.

All primary data (phylogenetic, qPCR, and phenotypic) and pipelines for statistical analysis and data visualization are available at https://github.com/zamanianlab/Bm-GAR-ms.
