# Supplementary material for: Pharmacological Profiling of a Brugia malayi Muscarinic Acetylcholine Receptor as a Putative Antiparasitic Target
Source: Antimicrob Agents Chemother. 2023 Jan 5;67(1):e01188-22. doi: 10.1128/aac.01188-22 (PMC9872666; doi:10.1128/aac.01188-22)
Supplement: Supplemental file 1 — Supplemental material. Download aac.01188-22-s0001.pdf, PDF file, 0.3 MB [file aac.01188-22-s0001.pdf]

Supplemental Material

All supplemental data and pipelines for analysis and data visualization are available at https://github.com/zamanianlab/Bm-GAR-ms .

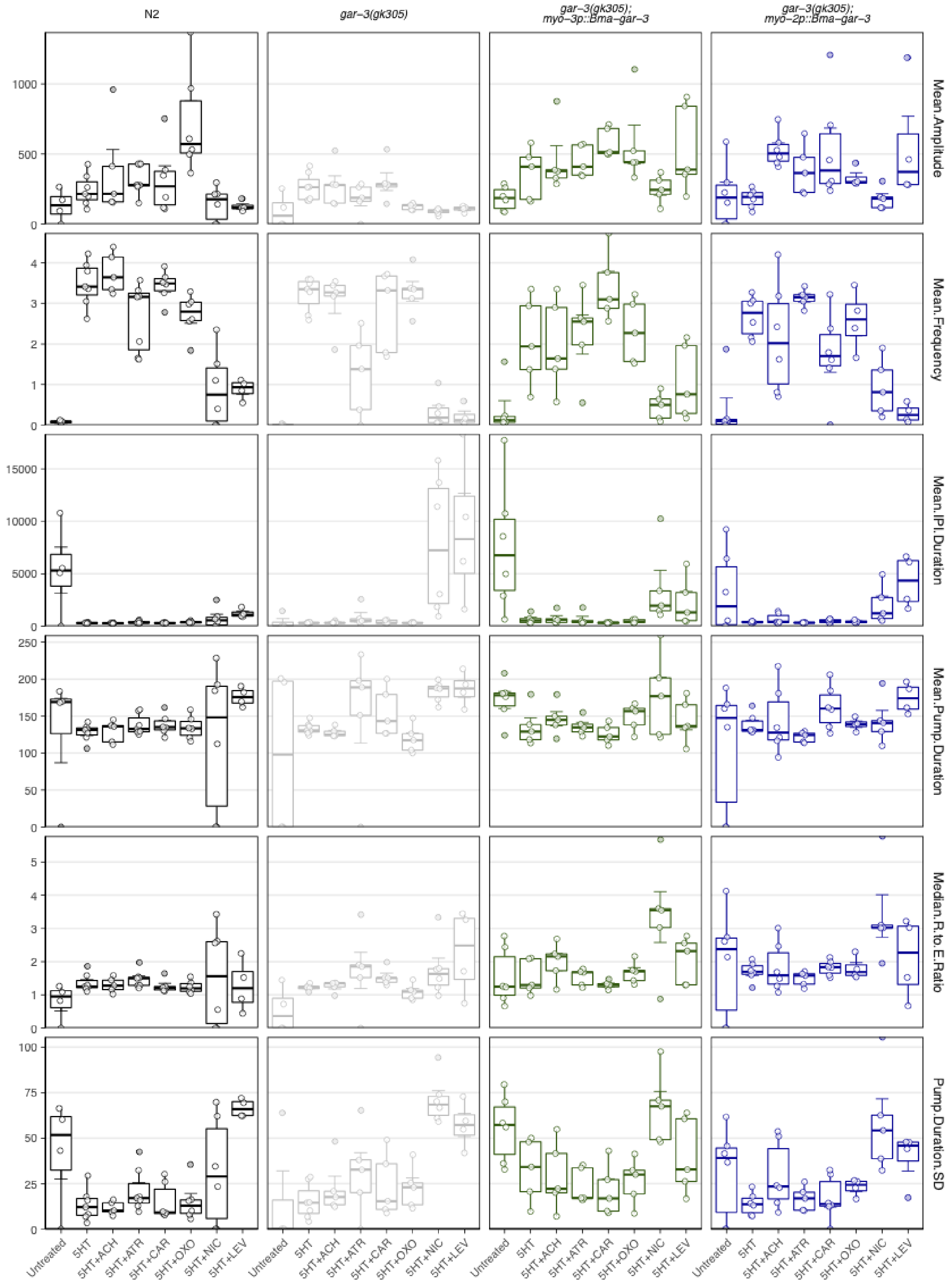

N2
gar-3(gk305)
gar-3(gk305); myo-3p::Bma-gar-3
gar-3(gk305); myo-2p::Bma-gar-3
Mean.Amplitude
Mean.Frequency
Mean.IPI.Duration
Mean.Pump.Duration
Median.R.to.E.Ratio
Pump.Duration.SD
Untreated
5HT
5HT+ACH
5HT+ATR
5HT+CAR
5HT+OXO
5HT+NIC
5HT+LEV

Supplemental Figure 1. Effects of cholinergic treatment on electrophysiological phenotypes measured using EPG recordings. Duration measures are reported in ms, amplitude = μV, and frequency = Hz.

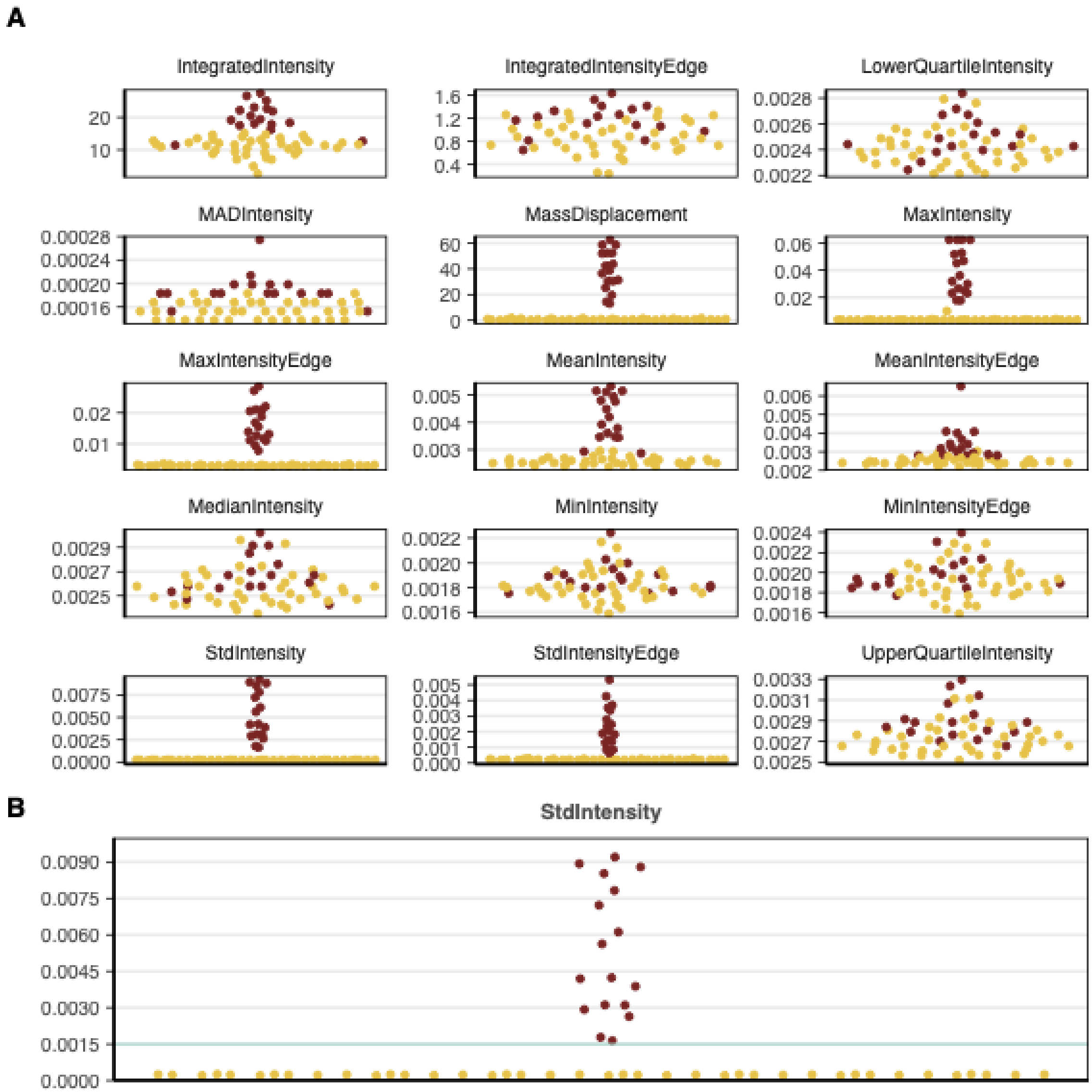

Supplemental Figure 2 A) Data was manually annotated by visually labeling worms in wrmXpress output images as GFP+ or GFP- (transgenic strain populations created with extrachromosomal arrays contain both GFP+ and GFP- populations indication expression of transgenes). Each GFP-related measure was plotted to determine a metric for filtering data for only GFP+ (transgene expressing) worms. B) StdIntensity, used to filter GFP+ worms from mixed populations, shows a clear separation between GFP+/- populations at a threshold of .0015.
